# Against Empathy?

**DOI:** 10.15694/mep.2018.0000213.1

**Published:** 2018-09-17

**Authors:** Trevor Thompson

**Affiliations:** 1 University of Bristol

**Keywords:** Empathy, sympathy, compassion

## Abstract

This article was migrated. The article was marked as recommended.

It is taken as a self-evident truth that it is a good thing for medical students and practitioners to develop and exhibit empathy in their clinical encounters. In 2016 the psychologist Paul Bloom published a work of popular psychology entitled, provocatively, “Against Empathy. The Case for Rational Compassion”. In this book he takes a strong line against empathy, arguing that it is not only not useful, but positively detrimental to human progress. Empathy, in the way Bloom defines it, leads to biased, shorted-sighted an practically useless action. In this essay I enjoy flirting with this broadside attack on one of our sacred cows. But I also a discover a major mismatch between what educationalists typically think of as empathy and the version presented by the author. The essay reviews this complex definitional landscape, visiting terms like pity, sympathy, moral imagination and compassion as well as the major varients of empathy itself. If we are going to avoid some of the evident pitfalls of emotion-dominated empathic responses, it really does help to be clear on what it is we have in mind when we discuss, teach and practice empathy.

## Point and Counterpoint on the Theme of Empathy in Healthcare Education

### Against Empathy?

A rare literary event occurred in my neighbourhood this year. A bookshop opened. I wandered in, was greeted by a congenial bookseller, and walked out with eight books. One of my spontaneous purchases was a work of popular psychology called *Against Empathy* by Paul Bloom. ( Bloom, 2018) Clever. I bet most people buying this book consider themselves empaths, and therefore see it part of their empathic duty to try and view the world through this set of contrarian lenses. Though I have always felt aligned to the concept of empathy I have, if I am really honest, never quite understood what it is and how it relates to cousins like sympathy and compassion.

Bloom kicks off by outlining the popularity of his quarry. You get, for instance, over 3000 returns on searching Amazon books for the term “empathy”. Higher ranking examples include “The Empathy Gap, Why Empathy is Essential”, “The Empathy Instinct: How to Create a More Civil Society” and “The Roots of Empathy, Changing the World Child by Child”. Websites include one listing everything Barack Obama ever had to say on the subject including an often-quoted statement that “The biggest deficit we have in the world..is an empathy deficit”.

Blooms takes the view that all this empathy isn’t just not good, but an overall harm to the progress of society. To understand this view you need to understand his definition - empathy is the “act of coming to experience the world as you think someone else does”. Empathy by this definition is actually/literally feeling another’s pain. We are reminded of the science of “mirror neurones” - cells that fire when we feel or do, but also when we *watch someone else* feel or do the same thing. ( lacoboni, 2009) A neural substrate for empathy in this Bloomsian sense.

And why bad? Because when we act on the basis of vicarious sensation we act narrowly, we act with bias and we act without reason (hence the book’s subtitle “the case for rational compassion”). He has examples. After the fatal shootings at Sandy Hook, Connecticut, in 2012, this affluent town was overwhelmed with children’s gifts often donated by poor people. He argues people felt the pain of the community and acted irrationally in response (the toys were no help). We feel, he says, much more the pain of those with whom we can identify and thus ignore the pain of those with whom we can’t. Empathy is innumerate and lacking in any long-term view.

Also empathy is bad for the empath - it can lead to burn out and make us less useful in responding to need. This is a major issue in the caring professions. Whilst many decry the documented (but disputed) erosion of empathy scores as medical students progress through the curriculum, I’ve wondered if this might be a *maturing*, rather than a *hardening* of the heart, as students learn to stop responding emotionally to the pageant of human suffering. ( Smith, Norman and Decety, 2017)

But while the book’s argument is enjoyably debunking, it is also chaotic. Bloom excludes from his critique what is elsewhere defined as *cognitive* empathy. This is not so much *feeling* the pain of the other but *understanding* it. Whilst we can feel moved by the plight of the patient, much better that we try and understand that plight with the hope of being able to respond usefully to it.

There are two big barriers to doctors understanding patients. First, doctors come mainly from high income backgrounds and most patients (especially in hospitals) come from low income backgrounds. Second, most patients are sick, and most doctors are well - and sick is another country. There are at least two trusted ways of crossing this frontier - really listening to what patients have to say and engaging with the arts (film, theatre, literature, visual arts etc) where alien perspectives are defined and refined, above the cognitive noise of the hospital.

For instance, here in Bristol Medical School we have a decade’s experience in putting to work extracts of the play *Cancer Tales* by Nell Dunn ( Dunn, 2002) - performed and directed by senior medical students for their junior colleagues. Here I explain how we justify “Theatre in the (Lecture) Theatre”:

The value of theatre in medical education is multi-faceted. There is the genius of the playwright in capturing the extraordinary in the commonplace, the skill of the director to interpret this meaning for a fresh audience and the courage of the cast to bring this new world to life on the stage. Through theatre we see behind the social mask to the agonies and ecstasies of the inner life, without leaving anybody emotionally exposed (except perhaps the audience). The play delivers a condensed and considered narrative that is so often missed, suppressed or drowned in the noise of real-world clinical encounters. ( Peterkin and Brett-MacLean, 2016)

Yes of course great art makes us *feel* but, especially in an educational context, we reflect on *why* it makes us feel as we do. For instance, one of the *Cancer Tales* narratives features a mother caring for a daughter with leukaemia. Here a student responds with both affective and cognitive empathy “so moving..the daughter had an acceptance and it seems much harder to witness someone dying than to be the one that dies”, “the carer wished to fix everything, but couldn’t”.

Bloom’s book, which has a lot (of mainly negative things) to say about moral implications of empathy, doesn’t reference the idea of “moral imagination”. This term from moral philosophy signifies the lofty ethical work of extending oneself “beyond the barriers of private experience and momentary events”. ( Birzer, 2015) In other words, moral perceptions don’t only derive from principles but from the human ability to *imagine* situations we hope to never actually encounter in our private lives. No amount of psychology will convince me that it isn’t a good thing to try and imagine the diverse, perverse and poignant predicaments to which we humans are prone. And such imaginative work feels a more serious undertaking than a simple reflex response to some witnessed or reported atrocity - something we set out to do rather than something that just happens.

But what of our basic emotional responses? Did a patient ever make you cry? I recall some moments as a junior doctor. Like watching a bewildered man in his thirties cradle his wife at the moment of her death in a side ward of a London hospital. Though the occasions are few, tears at such times feel absolutely OK, evidence that we are indeed human. To be honest I wish I could cry a bit more. But a tear is different to a prolonged weeping fit, and should only enhance our willingness and our ability to be helpful. So, I am a fan of a certain amount of emotional empathy and a relatively larger amount of (trained) cognitive empathy.

I was surprised that Bloom didn’t spend more time putting the boot into empathy’s semantic cousins, pity and sympathy. They are certainly unfashionable responses. Pity conveys the sense that the pitied person’s situation is truly awful but somewhat removed from the lifeworld of the pitier. I would not be likely to pity my daughter. The pitier, it seems, is in a much better situation than the pitied, so there is a danger of pity slipping into condescension. In any case the pitied is helpless to put things right of their own accord. Sympathy, a term coined by Scottish philosopher of the Enlightenment, Adam Smith, is the same thing as Bloom’s “emotional empathy” but with a sense too of the sympathiser directly expressing their sense of sorrow for the victim who, one feels, has fallen upon their difficulties through no fault of their own. Pity and sympathy are things deserved. Where Bloom attacks these types of fellow-feeling, layered with biases, and mainly emotional in tone, I find myself persuaded.

This brings me to compassion. Compassion, by my definitional system, is more than empathy (in fairness, Bloom makes a similar distinction, though definitions remain vague). Where empathy (both emotional and cognitive) triggers helpful action we have compassion. It can of course trigger other types of action. For instance, if I feel your pain I might want to run like hell and if I really understand what makes a man tick I might use that knowledge to manipulate him. It is said (think Hannibal Lecter in *Silence of the Lambs*) that psychopaths have an abundance of cognitive empathy but no emotional empathy - they get you then they gut you. But where empathy makes us act thoughtfully we have compassion - my favourite definition of which is “intelligent kindness”.

I am glad I picked up this book. It has helped me better define some very important terms, reminded me of the futility of many emotionally driven responses, and introduced me to some interesting psychological experiments. For instance, subjects primed to be empathic were more likely than controls to bump a child up a waiting list of similarly deserving children, proving for Bloom the moral vacuity of empathy-based action. ( Batson *et al.*, 1995) But the narrowness of Bloom’s definitional scope, excluding the imaginative and cognitive aspects of empathy, mean his conclusions are, at best, overstated, and I sallied forth with most of my original preconceptions safely intact. It is however a very good exercise to lead one’s sacred cows to the slaughter from time to time.

## Take Home Messages

• Empathy is a complex construct with both emotional and cognitive aspects
• Emotional empathy, akin to pity and sympathy, can lead to heavily biased responses
• Cognitive empathy can be developed and is more likely to promote understanding

## Notes On Contributors

Trevor Thompson is Professor of Primary Care Education in the University of Bristol. He is currently engaged in wide-ranging reform of medical education at Bristol, heading up work on cross-cutting helical themes including “Whole Person Care” and “Arts and Humanities”. His passion is for educational interventions that inform, challenge and inspire.

## References

[ref1] Batson C. D. Klein T. R. Highberger L. and Shaw L. L. (1995) Immorality from empathy-induced altruism: When compassion and justice conflict. Journal of Personality and Social Psychology. 68(6), pp.1042–1054. 10.1037/0022-3514.68.6.1042

[ref2] Birzer B. J. (2015) Russell Kirk: American Conservative. University Press of Kentucky.

[ref3] Bloom P. (2018) Against Empathy: The Case for Rational Compassion. S.l.: Vintage. 10.1097/TA.0000000000002071 30216236

[ref4] Dunn N. (2002) Cancer tales: true stories. Charlbury [Oxfordshire]: Amber Lane Press.

[ref5] Iacoboni M. (2009) Mirroring People: The Science of Empathy and How We Connect with Others. Original edition. New York: Picador USA.

[ref6] Peterkin A. and Brett-MacLean P. (eds) (2016) Keeping Reflection Fresh: A Practical Guide for Clinical Educators. Kent, Ohio: Kent State University Press.

[ref7] Smith K. E. Norman G. J. and Decety J. (2017) The complexity of empathy during medical school training: evidence for positive changes. Medical Education. 51(11), pp.1146–1159. 10.1111/medu.13398 28884471 PMC5657391

