# Supplementary material for: Against Empathy?
Source: MedEdPublish (2016). 2018 Sep 17;7:213. [Version 1] doi: 10.15694/mep.2018.0000213.1 (PMC10704415; doi:10.15694/mep.2018.0000213.1)
Supplement: Peer review history

# Reviewer report: Reviewer response for version 1

**DOI:** 10.21956/mep.19677.r29389

**Published:** 2019-07-09

**Authors:** Gary D. Rogers

**Affiliations:** 1 Griffith University

This review has been migrated. The reviewer awarded 4 stars out of 5 Thanks, Trevor, for this thought-provoking opinion piece. We have been interested in the somewhat broader concept of ‘human capabilities’. This idea includes the cognitive element of empathy as outlined in your piece, but explicitly explores the things that the coming machine revolution in medicine will not (at least not for a long time) be able to do better than human practitioners. We’ve found that is useful to think of these things in relation to all three of the *other* Bloom’s (Benjamin, 1956) domains. You’ve mentioned the cognitive elements and alluded to ‘trainable’ skills, which might be considered to reside in the psychomotor domain. You have also distinguished the emotional dimensions, which would reside in the affective domain, but arguably there is also a sense in which exploration of the experience of emotion during (real and simulated) clinical encounters during training shapes the formation of the values that underpin the health professions, under the Cruess’ idea of the ‘social contract’ (2000). Values also lie in the affective domain and we would argue are the critical piece of the ‘empathy puzzle’. You mentioned the ‘moral dimensions’ briefly in one paragraph in relation to the idea of ‘moral imagination’ but not explicitly the acquisition of a set of values that aligns with that of the profession for which an individual is training. I wonder if the idea of ‘human capabilities’, explicitly couched across all three domains of learning, enables us to tie together some of the concepts you have explored.

Reviewer Expertise:

NA

No decision status is available

# Reviewer report: Reviewer response for version 1

**DOI:** 10.21956/mep.19677.r29392

**Published:** 2018-09-27

**Authors:** Tan Nguyen

**Affiliations:** 1 Deakin University

This review has been migrated. The reviewer awarded 4 stars out of 5 This opinion piece is an excellent reminder to always challenge ourselves from the devils' advocate point of view. By doing so, particularly around the well-known acceptance of empathy being integral to good clinical practice, we can activate our life-long learning principles. That fundamentally is key to enhancing medical education practice.

Reviewer Expertise:

NA

No decision status is available

# Reviewer report: Reviewer response for version 1

**DOI:** 10.21956/mep.19677.r29394

**Published:** 2018-09-19

**Authors:** P Ravi Shankar

**Affiliations:** 1 American International Medical University

This review has been migrated. The reviewer awarded 4 stars out of 5 This is an interesting review of a controversially titled book. As we have seen in earlier papers, empathy is a concept which is often difficult to define and there are many aspects to empathy. Empathy may sometimes make one act in the heat of the moment but if empathy can be used to guide careful reasoning then it could result in a better decision than the one taken without empathy. Emotional empathy may need to be combined with cognitive empathy and careful thought to ensure that a rational decision is taken.

Reviewer Expertise:

NA

No decision status is available

# Reviewer report: Reviewer response for version 1

**DOI:** 10.21956/mep.19677.r29393

**Published:** 2018-09-17

**Authors:** Ian Wilson

**Affiliations:** 1 International Medical University

This review has been migrated. The reviewer awarded 5 stars out of 5 I would like to thank Prof Thompson for a challenging review of one of the major tenets of clinical practice - empathy. Like him I have wondered and reflected on empathy. It is a good thing, but it can lead to significant issues such as burnout. Like him I have modified my view of empathy as I have reflected on my practice.This is an extremely valuable piece of writing that should challenge teachers to really think about what they teach.

Reviewer Expertise:

NA

No decision status is available

# Reviewer report: Reviewer response for version 1

**DOI:** 10.21956/mep.19677.r29391

**Published:** 2018-09-17

**Authors:** Trevor Gibbs

**Affiliations:** 1 AMEE

This review has been migrated. The reviewer awarded 4 stars out of 5 A very well written personal piece which comes across as being written with deep concern regarding how we use certain words without thought. I can remember many years ago chatting to my GP trainee about the words empathy, sympathy, sharing and compassion and thinking that I had done a good job. Sadly the next day I found I had probably not as the trainee rushed into my room in floods of tears. She had tried to be empathetic rather than sympathetic and found that it had left her so emotionally drained that she just couldn't carry on with her clinic !! We do, I believe treat these words superficially, often not wishing to go as deep into / share our patients feelings because we know it might hurt, usually ourselves.After reading this paper I now need to go and buy the book to see if I can understand the words any better,but I believe I will be left with similar confusion and sometimes disagreement, but that's sometimes good.

Reviewer Expertise:

NA

No decision status is available

# Reviewer report: Reviewer response for version 1

**DOI:** 10.21956/mep.19677.r29390

**Published:** 2018-09-17

**Authors:** Hedy S. Wald

**Affiliations:** 1 Warren Alpert Medical School of Brown University

This review has been migrated. The reviewer awarded 4 stars out of 5 Thank you Dr Thompson for this engaging and scholarly piece on the challenges (and potentials?) of empathy…encouraging us to not use this term lightly. This article highlights the need for us to critically reflect on what we actually mean by “empathy” and whether we are able to being a necessary responsible, nuanced approach to empathy within medical education and practice…For the good of our patients, for our good as well. Let’s be “mindful” of the terms we use and how we operationalize them. Preserving empathy for humanism in medicine is a dominant theme in healthcare today and with good reason.I enjoyed the introduction and visual image of the book store, engaging the reader with story. The inclusion of the use of theatre is appreciated as is the thoughtful approach to terms such as sympathy and compassion.The paper may be strengthened with inclusion of some key papers in the field, namely Halpern’s “What is Clinical Empathy?” (2003, Journal of General Internal Medicine) helping to integrate cognitive and emotional lens for empathy (not binary) as well as Kumagai’s piece in Academic Medicine (2008) discussing the empathic response as both affective and cognitive with the “emotional response coupled with a cognitive awareness of oneself and others as separate, independent entities.” I have published on reflective writing as a potential means of cultivating “empathic humility” (Wald, 2011, pg. 362, Literature and Medicine) as I considered Macnaughton’s raising doubt about whether empathy “in the sense of emotional identification” is possible (“The Dangerous Practice of Empathy”, Lancet, 2009). Bringing a musical “angle,” I wrote that Alanis Morrissette’s song lyrics “Isn’t it ironic, don’t ya think,” resonated for me with the way a comment such as “I understand how you feel” within a clinical encounter could be deemed an empathic failure!A few other references that may be helpful: Garden’s “Expanding Clinical Empathy – An Activist Perspective” (JGIM, 2009) and hot off the press, Wald et al, 2018, Medical Teacher (“Grappling with Complexity” –Medical students’ reflective writings about challenging patient encounters as a window into professional identity formation”) where we conducted qualitative analyses revealing “nuanced empathy” for 3rd year medical students in challenging scenarios not empathy decline per se, an encouraging finding. So I would challenge “empathy as bad for the empath” and not necessarily leading to burnout as we train on calibrating emotional response with appropriate distancing. Given your (and burgeoning) interest in combining medicine and humanities, you (and the readership) may find Donato’s piece (Academic Medicine, 2009) on “Rationing Empathy” valuable – I have enjoyed using a combined approach with medical students of narrative medicine and evidence-based medicine in contemplating empathy and other topics in medical education.Yes, the concept of “intelligent kindness” is relevant -reference for this (Ballatt and Campling’s book “Intelligent Kindness”). We need further research on the use of the medical or health humanities in cultivating and preserving empathy (and neuroscience findings will be intriguing) and this includes promoting our own resilience/wellbeing as health professionals in order to provide empathic care (eg reflective writing as a “resiliency workout” Wald et al, 2015, Academic Medicine). Further research on the use of reflective practice and/or emotional intelligence skills for calibration of empathy within professionalism to help preserve and foster humanistic care is of keen interest. So maybe not “sacred cow to slaughter” per se but to more of a rigorous Socratic challenge session ;-). Dr. Thompson’s thoughtful piece is a welcome and valuable addition to this discourse, raising key issues to guide future inquiry.

Reviewer Expertise:

NA

No decision status is available
